# Comparative diagnostic accuracy of RIPASA and Alvarado scores for acute appendicitis: A prospective diagnostic accuracy study

**DOI:** 10.1097/MD.0000000000050453

**Published:** 2026-08-28

**Authors:** Waleed Ahmad, Fajar Saqib, Binish Tahreem, Mohammed Hammad Jaber Amin

**Affiliations:** a Department of General Surgery, Lahore Medical and Dental College, Lahore, Pakistan; b Department of Medicine, Alzaiem Alazhari University, Khartoum, Sudan.

**Keywords:** acute appendicitis, Alvarado score, clinical scoring systems, diagnostic accuracy, prospective diagnostic accuracy study, RIPASA score

## Abstract

To compare the diagnostic accuracy of the Raja Isteri Pengiran Anak Saleha Appendicitis (RIPASA) and Alvarado scoring systems in patients with suspected acute appendicitis using histopathology as the gold standard. A prospective study was conducted from December 2024 to April 2025 involving 100 patients at Ghurki Trust Teaching Hospital, Lahore. Patients presenting with right iliac fossa pain were assessed using both scoring systems. A score of 7 or higher from the Alvarado scale and 7.5 or higher from RIPASA indicated a positive result. Histopathological examination functioned as the definitive diagnostic method to establish correct diagnoses. The calculation of diagnostic accuracy and predictive values, together with sensitivity and specificity, was performed for both systems. In 100 patients (66% men and 64% under 40 years), histopathology showed appendicitis in 90% of patients. The RIPASA score demonstrated higher sensitivity (90.00%, 95% confidence interval [CI]: 81.85–95.31) compared to the Alvarado score (76.67%, 95% CI: 66.64–84.94), whereas Alvarado showed higher specificity (70.00%, 95% CI: 34.75–93.33) than RIPASA (20.00%, 95% CI: 2.52–55.61). Both scores had high positive predictive values (RIPASA: 91.01%; Alvarado: 95.83%) and low negative predictive values (RIPASA: 18.18%; Alvarado: 25.00%). Overall diagnostic accuracy was 83.00% for RIPASA and 76.00% for Alvarado. The RIPASA scoring system was found to be more sensitive and overall more diagnostic than the Alvarado score, but this difference was not found to be statistically significant. Both scoring systems are still clinically useful, and low scores cannot be used alone to rule out acute appendicitis.

## 1. Introduction

Acute appendicitis represents a global surgical emergency, affecting about 1 in 7 people throughout their lifetime.^[1,2]^ The condition persists as a significant medical issue because it presents diagnostic obstacles and leads to serious problems when patients do not receive quick medical care. The frequency of appendicitis shows different patterns in Asian and African areas because population dietary practices, including high dietary fiber consumption, seem to minimize fecaliths and blockages in the appendix.^[2]^ Patients aged between 10 and 19 experience the most appendicitis cases, but recent research shows that the condition is affecting increasing numbers of patients ranging from 30 to 69 years old.^[3]^

Acute appendicitis is still a major burden in Pakistan, especially in resource-constrained situations where a late diagnosis may lead to complications such as perforation and peritonitis. Pakistani studies have reported different rates of incidence and outcome based on access to timely surgical care and diagnostic resources.^[4–6]^ Despite the enhancements of diagnostic modalities, clinical decision-making remains mostly reliant on clinical judgment since, in most centers, advanced imaging is not readily available.

The diagnostic method of acute appendicitis involved clinical evaluation in the past which produced negative appendectomies ranging from 15% to 30% based on studies.^[7]^ The diagnostic precision in acute appendicitis has improved through ultrasound and computed tomography scans yet these methods prove impractical due to their restricted accessibility and high costs in particular environments.^[8]^ Computed tomography scans are highly diagnostic of acute appendicitis with sensitivities of 90% to 98% and specificities of 91% to 99%, but their widespread use has been limited by the concerns of radiation exposure especially when used in the younger patients.^[9–12]^ By contrast, ultrasound has a radiation-free, operator-dependent modality with variable sensitivity (70%–85%) and specificity (80%–90%), although its diagnostic use is less effective in cases like retrocecal or perforated appendicitis.^[10–13]^ The availability and affordability of these modalities however, restrict their use in many developing nations, including Pakistan, and hence, clinical scoring systems are very important diagnostic tools.

Different clinical scoring systems have been developed to improve diagnostic precision and decrease the number of surgeries that are not needed. The Alvarado scoring system functions as a popular diagnostic approach in western healthcare facilities through the assessment of clinical signs together with physical examination results and laboratory test outcomes to determine acute appendicitis risk.^[12]^ (Table 1).

**Table 1:** Alvarado scoring system.

Parameters	Score
Migratory pain	1
Anorexia	1
Nausea	1
Tenderness in right iliac fossa	2
Rebound tenderness	1
Elevated temperature	1
Leukocytosis	2
Shift to left	1
Total score	10

Healthcare providers developed the Raja Isteri Pengiran Anak Saleha Appendicitis (RIPASA) scoring system specifically to diagnose appendicitis within Southeast Asian populations.^[14]^ The RIPASA scoring system contains extra diagnostic parameters that incorporate patient age and gender along with their symptom duration before presentation enhancing its accuracy over the Alvarado score^[15]^ (Table 2).

**Table 2:** RIPASA scoring system.

Parameters	Score
Male	0.5
Female	1
Age <40 yr	1
Age >40 yr	0.5
Pain in right iliac fossa	1
Migratory pain	0.5
Anorexia	0.5
Nausea/vomiting	1
Length of symptoms <48 h	1
Length of symptoms >48 h	1
Tenderness in right iliac fossa	0.5
Guarding in right iliac fossa	1
Rebound tenderness	2
Rovsing sign	1
Elevated temperature	2
Leukocytosis	2
Unremarkable urine analysis	1
Foreign nationality	1
Total score	17.5

RIPASA = Raja Isteri Pengiran Anak Saleha Appendicitis score.

The existing literature has shown that the diagnostic performance of the Alvarado and RIPASA scoring systems varies across different populations. The Alvarado score has shown sensitivity ranging from 53% to 88% and specificity from 30% to 80%, whereas the RIPASA score has demonstrated higher sensitivity (up to 90%) but variable specificity.^[14–16]^ These differences indicate that these scoring systems should be validated on a population-specific basis.

Scoring systems provide valuable advantages but the diagnostic challenge of appendicitis persists because its signs show wide variability and can lead clinicians to misdiagnose it as other acute abdominal conditions. In patients undergoing appendectomy, histopathological examination of the resected appendix remains the reference standard for diagnostic confirmation.

Clinical outcome studies between Alvarado and RIPASA scoring systems should be conducted to identify which evaluation method provides the best results for different patient groups. Therefore, this study aimed to compare the diagnostic accuracy of the RIPASA and Alvarado scoring systems using histopathological findings as the reference standard.

## 2. Methods

This research study was planned as a prospective diagnostic accuracy study between December 2024 and April 2025 in Ghurki Trust Teaching Hospital, Lahore, Pakistan. The Ethical Review Board of Ghurki Trust Teaching Hospital approved the study (eef: 2024/11/*R*-39), and informed consent was obtained by all the participants before inclusion.

Eligibility screening entailed patients with symptomatic right iliac fossa pain, which was suggestive of acute appendicitis. Inclusion criteria were that patients aged 15 years or older with clinical suspicion of acute appendicitis would be included. The exclusion criteria were patients with generalized peritonitis, appendicular mass, pregnancy, and those unwilling to participate.

**Figure 1.**
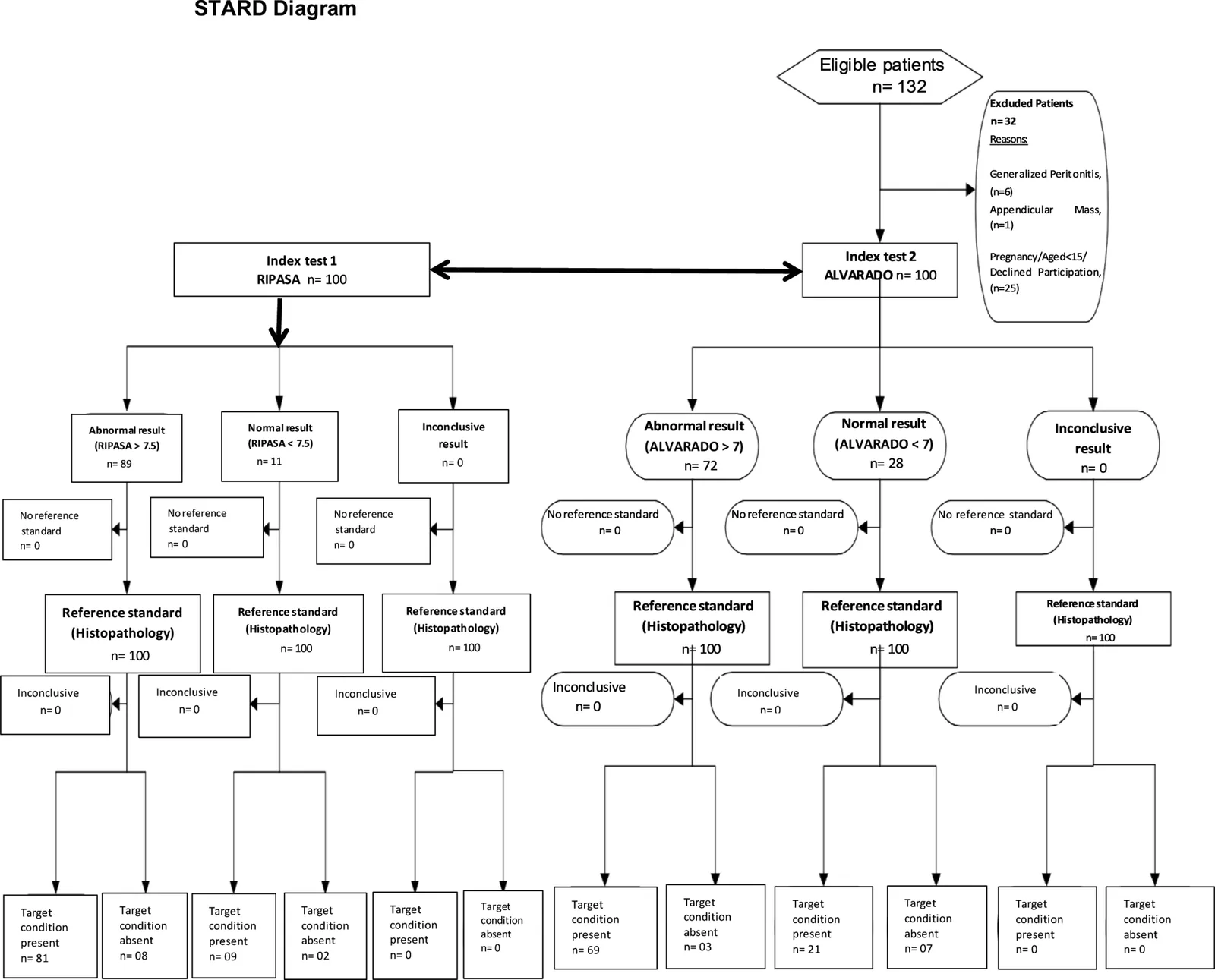
STARD flow diagram of participant recruitment, index testing (RIPASA and Alvarado scores), and reference standard outcomes (histopathology). RIPASA = Raja Isteri Pengiran Anak Saleha Appendicitis, STARD = Standards for Reporting Diagnostic Accuracy Studies.

As a result of these procedures, 132 patients who presented during the study period were screened, and 100 patients who met the inclusion criteria were enrolled, leaving a final sample size of 100 patients who underwent appendectomy (Fig. 1). A pragmatic precision-based method of diagnostic accuracy studies was used to determine the sample size. Previous literature has reported sensitivity of RIPASA scoring ranging between 85% and 90%.^[14,17,18]^ The estimated sample size was about 110 to 120 patients as per the calculation based on an anticipated 87.5% prevalence of histopathologically confirmed acute appendicitis, a 95% confidence interval (CI), a precision of 7%, and an assumed sensitivity of 85%, as reported in a previously published diagnostic accuracy study from Pakistan.^[19]^ Given the potential single-center design and the limitations of feasibility, 100 consecutive eligible patients were eventually recruited, which was deemed sufficient for the provision of reliable estimates of diagnostic accuracy. In our cohort, 90% of cases were found to have acute appendicitis on histopathology.

All patients were subjected to a standardized clinical examination, including a detailed history (migratory pain, anorexia, nausea/vomiting, and duration of symptoms) and physical examination findings (right iliac fossa tenderness, rebound tenderness, and guarding). The laboratory tests involved a complete blood count, with leukocytosis being defined as a white blood cell count exceeding 10,000/mm^3^. Selective cases were chosen for ultrasonography when the attending surgical consultant felt there was a clinical need for it. It was not a requirement for study inclusion, and the decision to proceed with surgery was not based on ultrasound alone, but rather on the overall clinical assessment. Computed tomography was not routinely used in the study population, was not readily available, and was not part of the usual diagnostic pathway.

Patients were initially examined by the surgical residents and later by senior surgical consultants. Two surgical residents (postgraduate year 2–4) under the supervision of a senior surgical consultant independently scored using both the Alvarado and RIPASA systems. The on-call consultant surgical team made the decision to proceed with surgical intervention based on overall clinical assessment, including history, examination findings, and lab results, where scoring systems were used as adjuncts and not as a primary decision-making tool.

The scoring based on the Alvarado and RIPASA systems was carried out at the time of admission before surgery to prevent incorporation bias. The clinicians who did the scoring were blinded with regard to histopathological results, which were only available after the operation and were not used to make decisions in the preoperative period. The Alvarado score used a cutoff value of ≥7 as originally proposed and widely validated in the literature, with the RIPASA score using a cutoff value of ≥7.5 based on the original derivation study by Chong et al.^[15]^ The scoring systems were used separately by the same surgical team and without cross-referencing between the Alvarado and the RIPASA scores to reduce assessment bias.

All patients had their appendectomy and the resected tissues were submitted to histopathology testing, which was used as the gold standard of diagnosis. Acute appendicitis was verified by the presence of neutrophilic infiltration of the appendiceal wall with or without other related features like perforation or gangrene. The patients were also followed up 7 days after surgery to determine the presence of any complications.

The research was carried out and reported in compliance with the Standards for Reporting Diagnostic Accuracy Studies guidelines.

### 2.1. Statistical analysis

Statistical Package for Social Sciences (SPSS) version 25.0 (IBM Corp.) was used to analyze the data. Continuous variables were expressed in the form of the mean standard deviation and the categorical variables in the form of the frequency and percentage.

The diagnostic accuracy of the Alvarado and RIPASA scoring systems was assessed by calculating sensitivity, specificity, positive predictive value (PPV), negative predictive value (NPV), and overall diagnostic accuracy with 95% CI.

To compare the diagnostic performance of the 2 scoring systems, the McNemar test was used to analyze the difference between paired proportions of correct and incorrect classifications. A *P* value of <.05 was considered statistically significant. There was no receiver operating characteristic analysis because the study was developed to measure prespecified clinically validated cutoff points and not to assess score performance with a range of different cutoffs.

## 3. Results

A total of 132 patients who were referred to the hospital with right iliac fossa pain were 1st evaluated regarding their eligibility. Among these, 32 patients were left out owing to predefined exclusion criteria which included generalized peritonitis, appendicular mass, pregnancy, and age below 15 years (Fig. 1). The ultimate study group was 100 patients who had undergone appendectomy and histopathological examination.

A total of 100 patients who required diagnostic assessments for possible acute appendicitis took part in the study. Male participants constituted 66% of those surveyed, while 64% were younger than 40 years old. The duration of symptoms in the majority of patients (76%) remained under 48 hours. The results showed acute appendicitis through histopathological examination in 90% of patients, but no appendicitis findings in the remaining 10%. The research data showed that 72% of patients had an Alvarado score of ≥7, while 89% met or exceeded a RIPASA score threshold of ≥7.5, corresponding to the predefined diagnostic cutoff values for the respective scoring systems. Ultrasonography was performed in 75 patients based on clinical indication. The results were suggestive of acute appendicitis in 48 patients, normal in 17 patients, and inconclusive in 10 patients. Ultrasonographic findings were evaluated in conjunction with the overall clinical evaluation, but were not used as a sole criterion for surgical removal of the appendix. The most common presenting symptom was right iliac fossa pain (100%), followed by rebound tenderness (83%), guarding (86%), nausea/vomiting (73%), migratory pain (67%), anorexia (62%), and fever (80%). Leukocytosis was present in 79% of patients (Table 3).

**Table 3:** Baseline and clinical characteristics of patients (N = 100).

Parameter	Category	Frequency, n (%)
Gender	Male	66 (66%)
	Female	34 (34%)
Age	<40 yr	64 (64%)
	>40 yr	36 (36%)
Duration of symptoms	<48 h	76 (76%)
	>48 h	24 (24%)
Clinical features used in scoring systems	Migratory pain	67 (67%)
	Anorexia	62 (62%)
	Nausea/vomiting	73 (73%)
	Right iliac fossa tenderness	100 (100%)
	Rebound tenderness	83 (83%)
	Guarding	86 (86%)
	Fever/elevated temperature	80 (80%)
Laboratory findings	Leukocytosis (>10,000/mm^3^)	79 (79%)
Ultrasonography performed	Yes	75 (75%)
	No	25 (25%)
Histopathology	Acute appendicitis positive	90 (90%)
	Acute appendicitis negative	10 (10%)
Alvarado score	>7	72 (72%)
	<7	28 (28%)
RIPASA score	>7.5	89 (89%)
	<7.5	11 (11%)

RIPASA = Raja Isteri Pengiran Anak Saleha Appendicitis score.

As per the research, the sensitivity measurement for the Alvarado scoring system reached 76.67% (95% CI: 66.64%–84.94%), and its specificity reached 70.00% (95% CI: 34.75%–93.33%). PPV reached 95.83% (95% CI: 89.27%–98.45%), but the NPV maintained only 25.00% (95% CI: 11.46%–43.40%). Research demonstrated that the Alvarado score yielded a diagnostic accuracy rate of 76.00% (95% CI: 66.43%–83.98%), with positive and negative likelihood ratios having a value of 2.56 and 0.33 respectively (Table 4).

**Table 4:** The Alvarado grading system’s diagnostic efficacy.

Alvarado/parameters	Histopathology positive	Histopathology negative	Total
>7	69	03	72
<7	21	07	28
			100

Statistic	Value	95% CI	
Sensitivity	76.67%	66.64–84.94	
Specificity	70.00%	34.75–93.33	
Positive likelihood ratio	2.56	1.02–6.42	
Negative likelihood ratio	0.33	0.21–0.53	
Positive predictive value	95.83%	89.27–98.45	
Negative predictive value	25.00%	11.46–43.40	
Accuracy	76.00%	66.43–88.98	

CI = confidence interval.

Histopathological confirmation showed that the prevalence of acute appendicitis in the study cohort was 90%. The diagnostic capability of the RIPASA scoring system achieved superior results, as it demonstrated 90.00% sensitivity (95% CI: 81.85%–95.31%) alongside 20.00% specificity (95% CI: 2.52%–55.61%). The PPV result for RIPASA reached 91.01% (95% CI: 83.08%–95.95%), but this high value came with a low NPV of 18.18% (95% CI: 2.28%–51.78%). The analysis of diagnosis with RIPASA revealed 83.00% (95% CI: 74.18%–89.77%) overall diagnostic accuracy that showed a positive likelihood ratio of 1.13 and a negative likelihood ratio of 0.50 (Table 5).

**Table 5:** The RIPASA grading system’s diagnostic efficacy.

RIPASA/parameters	Histopathology positive	Histopathology negative	Total
>7.5	81	08	89
<7.5	09	02	11
			100

Statistic	Value	95% CI	
Sensitivity	90.00%	81.85–95.32	
Specificity	20.00%	2.52–55.61	
Positive likelihood ratio	1.13	0.81–1.56	
Negative likelihood ratio	0.50	0.14–1.83	
Positive predictive value	91.01%	83.08–95.95	
Negative predictive value	18.18%	2.28–51.78	
Accuracy	83.00%	74.18–89.77	

CI = confidence interval, RIPASA = Raja Isteri Pengiran Anak Saleha Appendicitis score.

McNemar test demonstrated that the difference in diagnostic performance between the RIPASA and Alvarado scoring systems was not statistically significant (*P* = .07).

Follow up at 7 days showed few complications, with wound infection being observed in approximately 3% of patients, and no major complications such as intra-abdominal abscess or reoperative procedure reported.

## 4. Discussion

Although medical science has advanced its imaging and laboratory diagnostic capabilities, surgeons still find diagnosing acute appendicitis very difficult. The research used histopathological results as the benchmark to examine diagnostic accuracy levels of these systems among patients requiring acute appendicitis evaluation.

Research done in India involving 206 subjects during 2014 confirmed that the RIPASA score offered better sensitivity and specificity results.^[20]^ Research in India revealed that the Alvarado score had 64.44% sensitivity and 58.82% specificity, but the corresponding numbers for the RIPASA score were 87.78% and 76.47%.^[21]^ The 2018 Nepalese research established that the RIPASA score demonstrated 88.1% sensitivity coupled with 28.8% specificity, but the Alvarado resulted in 68.6% sensitivity with 28.6% specificity.^[22]^ Studies from Jordan, Kuwait, Iran, and Turkey, as well as a multicenter, cross-border study between Saudi Arabia and Egypt, corroborated the results of the original study in the Middle Eastern population as well.^[16,23–26]^

In general, the available evidence indicates consistency among the different populations, with the RIPASA score being more sensitive than the Alvarado score, and the difference in specificity was highly variable among the studies. The differences may be explained by differences in the socio-demographic characteristics of the patients, prevalence of the diseases, patient selection, clinical practice, and study methodology. The results of the present study are consistent with this general trend, as RIPASA showed greater sensitivity than the Alvarado score and estimated specificities similar to those reported in some earlier studies.

The results of the present study are in general agreement with the results reported in earlier studies conducted in South Asia and the Middle East, which further validates the diagnostic validity of the RIPASA scoring system in a population with similar clinical features. The sensitivity of RIPASA was 90.00%, notably higher than the 76.67% observed for Alvarado.

The specificity value of 70.00% in the Alvarado score exceeded that of RIPASA at 20.00%, yet its reduced sensitivity indicates missed identification of true appendicitis instances. Both diagnostic scores demonstrate a strong ability to detect true appendicitis cases, with positive predictive values reaching 95.83% for Alvarado and 91.01% for RIPASA. The negative predictive values of both scores are low (RIPASA: 18.18%; Alvarado: 25.00%), meaning low scores alone should not be relied upon to rule out appendicitis, specifically when operating in high-prevalence environments.

The fact that sensitivity and specificity differ across various studies could be attributed to a number of methodological and population-related factors. To start with, differences in patient demographics and disease spectrum, including variations in age distribution and severity at presentation, can impact the performance of clinical scoring systems. The sensitivity in studies with a higher percentage of early or atypical appendicitis cases may be lower because of less severe clinical manifestations. Second, scoring consistency can be influenced by inter-observer variability in the evaluation of clinical parameters such as rebound tenderness, guarding, and the Rovsing sign. Third, variability in the use of diagnostic thresholds may contribute to variation in reported sensitivity and specificity, since some studies involve multiple categories of cutoffs instead of a single binary cutoff. Further, disease prevalence plays a critical role in interpreting diagnostic performance; disease prevalence in settings (90% in the current study) can overinflate positive predictive values and deflate the reliability of specificity and negative predictive values. Finally, differences in clinical workflow, such as adjunct imaging and timing of assessment, can also further influence diagnostic performance. All these aspects underline the importance of contextual validation of scoring systems in different healthcare facilities.^[15,27,28]^

The wide confidence interval of the specificity of the RIPASA score (2.52%–55.61%) is suggestive of the low number of true negative cases in this study and limits the accuracy of this estimate. Thus, care should be taken in the interpretation of specificity findings.

The clinical application of the RIPASA scoring system could be useful because this score was more sensitive and overall more accurate than the Alvarado score in this cohort. While the difference in the 2 scoring systems was not statistically significant, RIPASA could be a valuable clinical tool for the evaluation of suspected acute appendicitis in a resource-limited area where advanced imaging is not readily available.

In spite of the fact that the RIPASA score was found to be more sensitive and overall more accurate than the Alvarado score, McNemar test was not able to demonstrate a statistically significant difference between the 2 systems. This implies that although RIPASA might offer better clinical sensitivity, the differences observed may not be an actual superiority in statistical terms. This can be at least partially explained by the small sample size and the small number of cases that were histopathologically negative, which decreases the statistical power of the studies to identify significant differences.

The evidence to support nonoperative management of carefully selected patients with uncomplicated acute appendicitis is increasing; thus, the role of clinical scoring systems will continue to evolve. As well as helping with the decision for surgical treatment, scoring systems like the Alvarado and RIPASA scores can be used to help determine which patients need more imaging, closer monitoring, or may be suitable for conservative treatment. However, the use of these scoring systems should be correlated with clinical assessment and not in lieu of sound clinical judgment.

The RIPASA score had better sensitivity, but the increased number of parameters compared to the Alvarado score might limit its practical use. The computational time involved in calculating RIPASA may be a limitation in a busy emergency environment. Moreover, certain parameters like guarding and rebound tenderness are susceptible to inter-observer variability, especially when measured by less experienced clinicians.

The 2 surgical residents used both scoring systems independently under the guidance of consultants, but inter-rater reliability was not statistically evaluated using Cohen kappa or similar statistics. This meant that the scores of different raters were not reproducible, and this could restrict the applicability of the results. Future multicenter studies could include a formal inter-rater reliability analysis to further confirm the consistency of these scoring systems across assessors.

Presence of parameters like foreign nationality might have restricted application in the current population where most patients are locals. Future research can examine how the RIPASA score can be adjusted to enhance its suitability for other populations. Thus, though RIPASA might be more sensitive, the simpler Alvarado score might still be preferable in an environment where fast decision-making is necessary.

This study has some critical limitations. Firstly, the high prevalence of appendicitis (90%) in the study population inflated the PPV and limited the reliability of the specificity and NPV. Secondly, the small number of negative histopathology cases (n = 10) reduces the statistical validity of specificity estimates. Thirdly, the absence of patients with generalized peritonitis and appendicular mass in the study could have led to spectrum bias. Fourthly, the clinicians who performed the scoring were blinded to the histopathological outcomes, but both the Alvarado and RIPASA scoring systems were used by the same surgical team. This might have caused an assessment bias that occurred during the assessment by the observer because knowledge of 1 assessment system may affect the other assessment system. Furthermore, formal inter-rater agreement was not assessed between the 2 resident assessors, possibly reducing the reproducibility of the results. Fifthly, despite the fact that the McNemar test was conducted, the study might have been underpowered and failed to detect any statistically significant differences due to the limited sample size and the low number of negative cases. Moreover, subgroup analysis according to age and gender was also not performed, which would have allowed obtaining more information about the score performance of different groups of patients. Also, the short follow-up time and the single-center study design limit the external validity of the results.

## 5. Conclusion

The RIPASA scoring system proved to have higher sensitivity and overall diagnostic accuracy than the Alvarado score, but without statistical significance. Both scoring systems continue to play a valuable role in the clinical assessment of suspected acute appendicitis, especially in resource-limited settings. Because it is more sensitive, the RIPASA may be valuable in identifying at-risk patients; however, the simpler Alvarado score may still be the preferred choice in fast-paced clinical settings. To identify conclusive superiority between the 2 scoring systems, larger multicenter trials are needed.

## Author contributions

**Conceptualization:** Waleed Ahmad.

**Data curation:** Waleed Ahmad, Fajar Saqib, Binish Tahreem.

**Formal analysis:** Fajar Saqib, Mohammed Hammad Jaber Amin.

**Methodology:** Waleed Ahmad, Binish Tahreem.

**Supervision:** Waleed Ahmad.

**Writing – original draft:** Waleed Ahmad, Fajar Saqib, Binish Tahreem.

**Writing – review & editing:** Mohammed Hammad Jaber Amin.
